# Beliefs and motives related to eating and body size: a comparison of high-BMI and normal-weight young adult women from rural and urban areas in Mexico

**DOI:** 10.1186/s12889-016-3695-4

**Published:** 2016-09-26

**Authors:** María C. Caamaño, Dolores Ronquillo, Riko Kimoto, Olga P. García, Kurt Z. Long, Jorge L. Rosado

**Affiliations:** 1 School of Natural Sciences, Universidad Autónoma de Querétaro, Querétaro, Mexico; 2 School of Public Health, University of Queensland, Brisbane, Australia; 3 Department of Epidemiology and Public Health, Swiss Tropical and Public Health Institute, Basel, Switzerland; 4 Av Ciencias S/N, Juriquilla, Querétaro, Qro. 76230 Mexico

**Keywords:** Obesity, Women, Eating habits, Beliefs, Culture, Social norms

## Abstract

**Background:**

Effective treatment and prevention of obesity and its co-morbidities requires the recognition and understanding of cultural and social aspects of eating practices. The objective of the present study was to identify social factors and beliefs that may explain undesirable eating practices among women with high body mass index (HBMI) compared with normal-weight (NW) women from rural and urban areas classified as middle-low socioeconomic status (SES) in the State of Querétaro, Mexico.

**Methods:**

A qualitative technique with individual in-depth interviews was used. Fifty-five women with either NW or HBMI from rural and urban areas participated in the study. The responses were analyzed by coding and grouping text fragments into categories in a data matrix, in order to make comparisons between BMI groups and between rural and urban women.

**Results:**

The habit of skipping breakfast prevailed among women with HBMI who also reported childhood food deprivation. Feelings related to eating seemed to be more important than losing weight among women with HBMI from urban and rural areas. Thus, overweight might be interpreted as a social symbol of the enjoyment of a good life, primarily in rural areas. Overweight was socially accepted when it occurred in children and in married woman, mainly because it is a symbol of the good life that the head of the household provides, and also because women may feel more relaxed about their weight when they already have a partner. The study also revealed that women with HBMI were not sufficiently motivated to lose weight unless they experience a physical indication of poor health.

**Conclusion:**

The findings from this study are helpful in the understanding of the reasons why strategies for the prevention and treatment of obesity may not be as effective as expected. The belief system of particular social groups within different SESs should be considered in order to understand the etiology of obesity and develop effective strategies.

## Background

Obesity is a biological disorder that has gained attention from health institutions due to the rapid increase thereof in recent decades and its health implications [1]. Mexico has been positioned among the countries with the highest prevalence of overweight and obesity in the world in 2013 [2]. With a view to resolving the problem, most strategies have been aimed at altering people’s food choices and increasing physical activity through public health education campaigns and policies that limit the availability of calorie-dense foods [3]. However, despite the efforts in mass media and the dedication of health professionals, there is still a high prevalence of obesity [4, 5]. Furthermore, there is an irregular spread of obesity across the population, being disproportionately high in the middle and middle-low socioeconomic population in Mexico [6]. Although public health institutions blame an obesogenic environment that prevails in the most industrialized areas, the prevalence of obesity is not only high in urban areas (34 %), but also in rural communities (27 %) [4], where the population is presumed to have a healthier lifestyle, due to their traditional food patterns and low levels of sedentary activity [7].

Several authors have recommended the exploration of the root causes of eating practices embedded in specific environments in order to develop effective customized strategies that consider social and cultural influences [8–10]. Some argue that perception of an obesity epidemic depends on the social and cultural values that shape collective conceptualizations of body weight and health [11–13].

In Mexico, few studies have explored certain cultural values and beliefs related to eating practices in different regions [14–16]. Most of them have drawn conclusions within a single population or in several similar populations, but it is important to identify the factors that predominate among people with obesity compared to normal-weight individuals. In addition, in consideration of the fact that urbanized environments facilitate an unhealthy lifestyle [17], it is important to confirm whether or not such factors differ among environments with different levels of urbanization.

This study was aimed at identifying social factors and beliefs that may explain unhealthy eating among middle-low-SES housewives who are responsible for planning and preparing the family’s food, with high level of overweight or obesity compared with normal-weight women. The study also compared already-identified factors between women living in rural areas and those living in urban areas.

## Methods

A qualitative research methodology based on grounded theory was used to address the objective of the study [18]. The qualitative technique utilized was individual in-depth interviews of women with apparent normal weight and with apparent obesity from rural and urban areas. The differentiation between normal weight and obesity, as well as rural and urban areas ensured maximum variation sampling in order to provide better insight into potential obesity-promoting factors [18].

Some factors associated with obesity in women from rural areas were identified previously. This study re-analyzed data from rural participants from a different perspective (focusing on beliefs and motives related to eating behavior and body size), and compared it to data from women living in an urban area. The detailed methodology of the study carried out in the rural area was previously described in [19].

### Participants and setting

A total of 60 housewives from 25 to 45 years of age were approached and recruited based on their estimated weight: 30 from two rural communities in the State of Queretaro, Mexico, in the municipalities of Colon and Tequisquiapan (population <2500; marginalization index four out of five), from which 15 had apparent obesity and 15 had apparent normal weight, and 30 from a marginal urban area at the north end of Queretaro with the same proportion of women with obesity and normal weight. The selected sample size ensured saturation according to previous reported studies. Both the rural and the urban area were identified as middle-low SES, in stratum 3 or 4 out of 7 according to the National Center of Geography and Informatics [20].

Women were excluded if they were following any weight loss intervention prescribed by a physician or nutritionist, or if they had a family member in their household that was already participating in the study. Women over 45 years of age were excluded because their body weight could be confounded by possible weight gain after menopause due to hormonal changes [21]. Women who met the inclusion criteria attended an informational meeting and agreed to participate after receiving oral and written information about the general purpose of the study and the procedures. Then, an appointment was scheduled for the interview at their homes. The study was approved by the Bioethics Committee of the *Universidad Autónoma de Querétaro*.

### Interview

A psychologist, trained as interviewer, in addition to one or two collaborators visited the participants at their homes in order to conduct the interview. All interviews were audio-recorded with prior authorization from the participants.

The topic guide included questions related to: foods they eat and the reason why, their perception about unhealthy and healthy foods, perception and knowledge about obesity, and the consequences of obesity.

After the interview, the women were measured and weighed to confirm their BMI classification [22].

### Analysis and interpretation

Each interview was transcribed verbatim and the text was segmented and assigned to emerging codes. Subsequently, codes were grouped into categories and sub-categories that formed a data matrix, on an Excel worksheet, which facilitated the making of connections and comparisons.

The data were interpreted by means of the constant comparative technique for grounded theory [23], comparing code and category data within and between the four groups formed by BMI and urbanization level. Our theory was developed by interpreting data that were consciously explained by the participants, such as eating habits or lifestyle; when approaching concepts related to motives, beliefs, and attitudes, our interpretation took into account the fact that the women may not have been conscious about such factors and also that they may lie or conceal a situation to enhance their self-esteem [24].

In order to validate conclusions and to reduce the researchers’ bias in the interpretation of data, the interviewer analyzed the coded data independently. After comparing her conclusions with those from the main analysis performed by two other researchers, only common conclusions were considered valid.

## Results

After measuring the participants, the women who were considered normal weight had a BMI < 25.9 kg/m^2^, and of the women who were estimated to be obese, four had a BMI < 30 kg/m^2^. Thus, the researchers decided to include the two overweight participants who still had a high BMI (HBMI) (≥27.8 kg/m^2^) and to exclude the other two participants whose BMI was closest to normal weight. This decision was made in order to account for most of the valuable gathered information, ensuring maximum variation sampling while still allowing comparisons to be made. One participant was excluded because the interview was interrupted, and two others were excluded because the researchers determined that there was possible bias due to the participant’s occupation or the influence of other persons during the interview. Thus, the responses of 55 women were analyzed, and the data matrix developed with emerging themes confirmed data saturation. All participants were married or living with their male partner, except one who was separated from her partner. The duration of the interviews was between 45 and 90 min. The socio-demographic characteristics of the participants are shown in Table 1. Based on the data, six main themes were identified as social factors and beliefs that may explain unhealthy eating among housewives; most of them differed between women with HBMI and normal-weight women, and few differed between rural and urban areas.

**Table 1:** Socio-demographic characteristics of the participants

	Rural context						Urban context				
ID	Age (y)	BMI (kg/m2)	Children (n)	Occupation	Education (y)	ID	Age (y)	BMI (kg/m2)	Children (n)	Occupation^a^	Education (y)
Participants with normal weight											
1001^b^						3001	25	24,8	5	Hw	6
1004	41	24,9	1	Hw^a^	6	3004	38	24,0	3	Hw	6
1006	32	24,8	1	Hw	6	3007	30	24,6	2	Hw & Housemaid	6
1009	28	24,2	2	Hw	4	3013	44	24,6	3	Hw	6
1011	27	24,8	1	Hw	9	3014	30	23,8	3	Hw & Stylist	6
1013	33	24,0	4	Hw	9	3015	30	23,6	2	Hw	6
1014	30	23,8	1	Hw	2	3018	26	24,1	2	Hw & Housemaid	6
1015	28	24,0	3	Hw	6	3019	40	24,8	2	Hw	6
1016	40	24,4	4	Hw	6	3021	30	24,5	3	Hw	9
2005	23	22,4	1	Hw	6	3022	33	23,9	1	Hw & Seller	9
2006	37	25,3	5	Hw	6	3024	34	23,2	2	Hw	0
2007	27	23,0	3	Hw	9	3025	40	25,0	3	Hw	6
2009	31	24,8	2	Hw	6	3026	42	24,9	3	Hw	4
2013	24	25,8	2	Hw	9	3028	26	24,6	3	Hw	9
						3030	40	24,7	3	Hw	9
Participants with overweight or obesity											
1002	28	31,7	0^c^	Hw	7	3002	31	30,6	3	Hw & Housemaid	7
1005	29	32,4	2	Hw	3	3003	33	37,3	2	Hw	11
1007	29	41,2	1	Hw	6	3005	28	37,0	2	Hw	6
1008	42	31,3	3	Hw	6	3006	39	34,9	2	Hw	6
1010	25	39,1	4	Hw	6	3008	32	31,7	2	Hw	6
1012	29	36,0	1	Hw	6	3009	35	29,8	2	Hw	7
2002	24	39,6	0	Hw	6	3010	39	41,9	2	Hw & Housemaid	9
2004	38	37,9	3	Hw	2	3012	33	33,2	2	Hw	6
2008	33	36,7	4	Hw	9	3016	31	27,8	2	Hw & Employee	7
2010	29	31,6	3	Hw & Store attendant	6	3017	28	33,7	4	Hw & Employee	6
2011	44	32,9	6	Hw	7	3020	36	30,3	5	Hw	6
2012	34	38,9	2	Hw	9	3023	44	35,7	2	Hw	6
2014	27	30,7	2	Hw	7	3027	35	39,8	2	Hw	6

^a^ Housewife

^b^ Lost to follow up, and was not measured nor answer additional questionnaires

^c^ Woman not living with partner anymore

### Eating practices

There were some differences in the reported regular meals between rural and urban women. Table 2 describes common diets in the rural and urban areas. In general, rural women always had maize *tortillas,*^1^ fried beans, and *nopales*^2^ available, and included them every day in one or two meals. Most rural women had two high-calorie meals during the day, the mid-morning meal and the mid-day main meal, the latter sometimes being so late that it is the last meal of the day. Most of the rural families ate at their homes to save money.

**Table 2:** Summary of reported habitual meals during the day by study groups

	Meal time				
	Breakfast	Mid-morning meal	Main meal	Dinner	Snacks
	8–10 am	10–1 pm	1–6 pm	6–9 pm	In-between meals
Rural context					
High BMI^a^	Nothing or a drink: coffee, milk or *atole* ^b^. Sometimes a piece of bread.	Potatoes, meat stew or eggs or fried beans *Tortillas* ^b^. Soda	Nothing if they ate late at mid-morning. Rice or soup Meat stew *Tortillas* ^b^. A side dish: mostly fried beans and/or *nopales* ^b^. Soda or fruit water.	Coffee A piece of bread or maize *tortillas* ^b^ with left-overs from lunch or beans	A piece of bread *Chicharrón* ^b^ *or churritos* ^b^ with lime and chilli sauce, Soda Fruit or vegetables (apple or cucumber).
Normal weight	Coffee, milk or *atole* ^b^. RTEC, or eggs, or a piece of bread.	Nothing or eggs with ham or sausages, or meat stew, *Tortillas* ^b^.	Soup or rice Eggs Fried beans (Meat dishes were not often.)	Left-overs from lunch, or RTEC, or a glass of milk.	Nothing Few ate fruit or *churritos* ^b^
Urban context					
High BMI^a^	Coffee or milk or *atole* ^b^. A piece of bread or sandwich or quesadillas or RTEC^b^ or “tacos” or fruit.	Eggs with beans, or grilled meat, or *nopales* ^b^, or a soup, or meat stew. Maize "tortillas". Or RTEC or sandwich.	Vegetable cream soup or pasta or lentils soup or rice. Meat stew or potatoes or broad beans stew. Sometimes hot dogs If eating out: Roasted chicken or *carnitas* ^b^.	Sandwich, or tacos If eating out: *tacos* ^b^ from a street stall.	Nothing or fruit, bread or *churritos* ^b^.
Normal weight	Fruit shake or coffee A piece of bread; or RTEC with milk or fruit or *tacos*	Nothing or meat stew or fried beans or eggs with *tortillas* ^b^. Or sandwich or fruit.	Vegetable cream soup or pasta or lentils soup or rice Meat stew or potatoes stew; Salads or boiled vegetables.	A glass of milk with a piece of bread or RTEC^b^ or fruit. Occasionally meat *tacos* ^b^.	Did not mention snacks.

^a^BMI ≥ 27.8 kg/m2

^b^ *RTEC* ready to eat cereal, most of the times were sugared flakes. *Atole*: flavored maize milk shake. *Tortillas*: thin, round, unleavened bread prepared from cornmeal, baked on a flat plate of iron. *Tacos*: tortilla filled with beans, vegetables or meat. *Nopales*: edible cactus (*Opunita sp*). *Chicharrón*: fried wheat flour piece. *Carnitas*: fried pork meat. *Churritos*: fried maize flour sticks

Women from the urban area had more variety in their diets; they did not include much edible cactus or vegetables, and they ate out more often, mainly *tacos*^3^ but also seafood or western foods such as pizza and roast chicken. Women from the urban area mentioned frequently that food had changed since their childhood. For instance, they ate fewer beans and less rice and edible cactus than they had done previously.

### Social determinants of eating habits

A regular habit that prevailed more among women with HBMI from rural and urban environments than among normal weight women was skipping breakfast or having a very small breakfast; e.g., a glass of milk. Most women assumed that this habit comes from their childhood, when they experienced food deprivation.

*I do not have breakfast. I do not like to have breakfast; because since I was with my mother, she never gave us breakfast. She never had enough money to give us breakfast. I think that is the reason for this habit. In my home I do give breakfast to my children but I do not have breakfast. Then they ask me “Why don’t you have breakfast?” And I say “You keep eating” and meanwhile I do my chores. [Rural obese-2012]*

Women who reported that they had the habit of skipping breakfast also reported frequently snacking during the morning or eating a large mid-morning meal. In the rural area, an active social life such as bringing lunch to their children at school, or visiting neighbors or relatives induced them to overeat in the mid-morning. In addition, excessive hospitality among rural woman was a social norm: casual encounters encouraged the hostess to share a meal accompanied by soft drinks, and the guest was socially compelled to accept whether or not she was hungry.

### Social cognition regarding high calorie food intake

Soft drink intake, mainly regular cola, was very common among women from urban as well as rural areas and in both BMI groups. Soft drinks were reported to be consumed at any time during weekdays or weekends, and during celebrations consumption thereof was mandatory. Most women justified their consumption, although their attitudes demonstrated their acknowledgement that soft drinks are not a healthy choice.

*(I drink)…if there is enough money, then a soda, and if there isn’t, then water, in the evening “atole” normally, I drink soda only at lunch, only at lunch. [Urban obese-3017]*

It was clear that soft drink consumption was a deep-seated habit that sometimes was perceived by participants as an addiction. The habit of drinking soft drinks was clearly associated with certain occasions in their social life, such as casual encounters, celebrations, or certain meals with a high-energy content, where soft drinks seemed necessary to enjoy the moment.

Cooking with oil and avoiding lard, which was considered harmful, was very common among rural women; they fried beans and edible cactus (*nopales*) regularly. Women with normal weight reported being more careful about the use of oil to prepare foods than HBMI women. All women from rural and urban areas acknowledged that consuming an excess of fat and oil was unhealthy, although they associated oil with good taste.

*I eat fried beans, with lots of oil, or any meal, but with lots of oil, and my glass of soda. I consider that the good and the bad (good for the taste, bad for her health). [Rural obese -1010]*

*Here, they (family) do not tolerate greasy food; if I give them greasy food, they tell me jokingly, “Don’t you have more oil?” Because it has happened that I overuse oil and they do tell me [Rural normal weight-2007]*

### Social meanings of eating

In general, women with HBMI from both rural and urban areas showed more enthusiasm than normal-weight women when talking about food choices or food preparation. This implies that enjoying food was significant for them. They associated eating well with feeling satiated, with no consideration of the nutritional value of the food; it seemed that spending less and becoming completely satiated was a smart decision. Perhaps as a result of these social meanings, some women with HBMI mentioned experiencing regret very often after eating too much. This was reported less frequently by normal weight women.

In addition, it was perceived that a decision to buy certain foods was triggered by the need to evidence certain status. Beans and edible cactus were not only perceived as healthy foods, but also as an ordinary meal for underprivileged people, thus the consumption of industrialized food, such as soft drinks or pre-packaged cookies could distinguish them from unfortunate people. Mostly HBMI women revealed feeling proud about overeating, possibly in order to demonstrate that they have a better SES than others. Women with normal weight from both areas did not mention allowing themselves to overeat when they had the opportunity to do so; instead, they frequently mentioned limiting the amount of food they consume.

### Motivation to improve eating behavior

Most HBMI women stated their intention to improve their eating behavior, and frequently mentioned that following a diet was the way to decrease body weight. However, most of them associated following a diet with their own bad experiences or those of acquaintances. Thus, participants referred to diets as a sacrifice that implied experiencing hunger, weakness, and emotional changes. Some women also reported having acquaintances who after finishing or quitting the diet, re-gained even more weight than before. Therefore, most HBMI women from urban and rural areas believed that it was not worthwhile to follow a specific diet; instead, some mentioned creating their own diets that consisted of avoiding one or two foods, such as soft drinks, breads, or tortillas. However, they indicated that they maintained the food restriction for a short period: less than 1 month. By contrast, normal-weight women did not mention following diets or restricting foods as often as did participants with HBMI. Some normal-weight women agreed with changing habits in the long term.

*Normally they ask me: What diet should I follow? (laugh), but it is not following a diet… It is not difficult, is the routine of each person… I see that it has to be like that, one’s willingness, one’s readiness, there has to be an effort to change, because sometimes they tell me “it is very hard, you (can do it because you) have the time” and it is not that I have the time, it is simple, buying healthier stuff. [Normal weight-urban-3015]*

All women with HBMI mentioned their intentions to improve their eating habits, however a valid motivation was recognized from women, either women with HBMI or normal-weight women who had experienced weight loss as a result of a personal effort. The only identified valid reason to change eating habits was a diagnosed disease that was painful or perceived as life threatening that they had personally or that a household member had. This suggests that losing weight is worthwhile only when a real possibility of death is perceived.

In addition, similar motives were identified in women with HBMI who expressed concern about physical indications of poor health, such as becoming fatigued or short of breath when walking a short distance, in addition to worries about the possibility of dying and leaving their young children defenseless. Such expressions were not observed among normal-weight women.

*At the medical facility I had some studies done, and they told me they (blood lipids) were high… The thing is I became unwell, I was dizzy everywhere, my arm was aching, and that was when I decided to exercise and eat healthier, for my disease and because my boy was too young. [Obese-urban-3003]*

Some participants admitted certain factors that discouraged them from changing eating habits. A feeling of resignation was perceived when they assumed that they were destined to be fat, particularly when they recognized the similarity of their body shape with that of their parents or sisters. This attitude was often brought to mind when they recalled frustrated past efforts to lose weight. Women became easily disappointed about making an effort to lose weight because of their high expectations of losing weight rapidly.

### Social meaning of body size

The final part of the interview focused on the perception of their own weight and other people’s weight. The most relevant finding was that overweight and even mild obesity were accepted or even preferred at certain life stages by almost all women regardless of their environment’s level of urbanization or their body weight.

An overweight child meant better health than did a normal-weight child. Several women with normal-weight children mentioned that their friends or relatives had recommended them to take the child to the doctor because they seemed malnourished. Despite the high prevalence of obese children in the area, only few normal-weight women showed concern about it. On the contrary, most women from rural and urban areas perceived healthiness in an overweight child.

*… and my daughter, I used to see her skinny skinny (laugh) like a stick, and I took her to several doctors and she was checked for everything, and in the end the pediatrician told me:” Leave that girl alone because she is fine, she needs nothing, she is not malnourished, she has no bugs or anything, she is fine.” [Normal weight-urban-3015]*

*(My daughter) has a very fat boy, he is 8 years old, he is blessed because he is very chubby. [Normal weight-urban-3014]*

When girls became adolescents, they started to be encouraged to take care of their body weight, since they needed to be appealing for men and be able to get married. Almost all women from both areas with HBMI and normal weight mentioned being much slimmer when they were single. Most women said that it was natural to gain weight when they get married or had children. Some believed that this fact was attributable to overeating during lactation, in order to ensure good nutrition for their newborns. Others reported focusing more on their children’s health than on their own well-being, and accepted that they were careless about their own eating habits. A few women indicated that they had no need for a good figure because they already had a husband.

*I have cousins, they are fat, chubby, my cousin that is single she says “if you compare yourself with me, then you are the one that has no children and I am the one that has them”. [Normal weight-urban-3015]*

We identified several social norms that led women to feel comfortable with extra weight. For married women, overweight meant that they are enjoying a good life, which involves eating well, being accepted and cared by their husbands, and having good health. Women with normal weight, who, compared to the majority of women are perceived as underweight, represented the opposite: they are either malnourished, mistreated by the husband, depressed, or ill. Several normal-weight married women expressed feeling criticized for their weight.

*…Once, my family made me feel bad, because they said “Well, what is wrong with you? Are you sick?, You look green, skinny, hollow-eyed, ….and we should take you to the doctor” They made me feel really bad. [Rural normal weight 1009]*

In the selected socioeconomic stratum, where the majority of women were overweight, the people who had lost weight were either women who became ill, mainly from diabetes, or who fell into depression due to marital conflicts. In fact, some women, mainly with HBMI, mentioned that they had lost weight at some time during emotionally stressful situations. Such perceptions may explain why losing weight is associated with adverse situations.

*Before, I was fat, now he (my husband) tells me “You have become really thin!” And I tell him “When I met you, I was very fat” and that’s it, well, not fat, he says I used to have broad legs and wide hips, then as you see all that ends with this disease (diabetes). Because I used to have a good body but when I got the disease then all of me got thin. But then, don’t you think that he (husband) doesn’t love me anymore. [Rural-Overweighted-2006]*

Other women mentioned losing weight when they were lactating; they even remembered being persuaded by relatives to stop lactation in order to stop losing weight.

*…I started then to consider myself very slim…my husband told me “Stop breastfeeding her, let’s go and buy her the canned milk, or let’s go (to the medical facility) for a recipe.” And that was when I started to recover [Rural Normal weight-2005]*

Some women from both urban and rural areas reported that their husbands encouraged them to improve their eating habits or to exercise in order to have good health. However, since single women were concerned about their weight in order to be attractive to men, a married woman who suddenly achieved normal weight might be socially discredited. Few women who had lost weight or intended to go out to exercise pointed out their husbands’ attitudes of jealousy or embarrassment with other men, because people might think the wife is behaving like a single woman.

*…because in fact, when I had my second baby, my grandmother used to warn my husband: “Be foolish and then she gets another (man), because she remained like a single lady, she doesn’t look like a mother.” [Normal weight-rural-2007]*

In urban as well as in rural environments, women in both BMI groups perceived an overweight woman as a symbol of good emotional status, good economic status, and, if no physical symptoms appeared, then also of good health.

## Discussion

The present study could validate previously-documented findings in rural women [19]. Moreover, it confirms that some factors also occur in urban areas. Women living in rural and urban areas shared similar beliefs and social norms although their eating practices differ due to restricted food availability in the studied rural zone. However, differences within rural and urban areas were identified between women with normal weight and HBMI.

Many participants with HBMI who experienced food insecurity in their childhood reported that they had carried over the habit of skipping breakfast, but they had increased snacks and foods in the mid-morning. Food insecurity, as well as skipping breakfast and having a high energy mid-morning meal, had been associated with obesity in other studies [25–27]. This study showed that past experiences of deprivation may be associated with a greater enjoyment of food. This was perceived in women with HBMI who also seemed to over-appreciate satiation, compared to normal-weight women. This finding agrees with those of Olson, Bove, and Miller [28], who also found that experiences of poverty-associated food deprivation in childhood appear to super-motivate some women to actively avoid food insecurity in adulthood. According to Epstein et al. [29], food reinforcement value increases with past experiences of food scarcity. Thus, childhood food insecurity may be an important social factor that leads to adult obesity by maintaining the habit of skipping breakfast and, when individuals become wealthy enough, overeating at other meal times.

Despite the sufficient knowledge about unhealthy foods, which has also been recognized in a similar population [30], most women reported drinking cola soft drinks regularly and some women indicated that they were addicted to it. The amount of cola soft drink intake could not be quantified in the interviews, but a previous study conducted in the same area found it to be clearly associated with obesity and body fat [27]. Some women also mentioned feeling regret after overeating. Overeating has been described as an altered feeding homeostasis, and it has been suggested that excess intake of palatable foods may be a problem of food addiction [31, 32]. However, there is still controversy regarding the existence of food addiction as a neuro-biological disorder or whether it is simply better described as a behavioral dependence [31]. In this study, insights into the social lives of participants with HBMI who self-reported overeating or food addiction, compared to normal-weight women, indicated that there may be stronger beliefs in their value system, than values regarding healthy eating or avoiding overeating in order to prevent diseases. For instance, feeling satiated may be more important than eating healthy; thus, low-priced energy-dense foods are preferred to reach satiation. Also, enjoying highly palatable foods was over-appreciated by participants with HBMI compared to normal-weight participants; this phenomenon had been previously noted in other populations [29].

Most women mentioned that eating inadequately was a social habit shared in casual encounters or parties; this suggests that overeating could also be the result of implicit social norms in medium-low SES groups. Ochoa [16] investigated eating habits in a similar SES community near Mexico City, and found that intake of certain foods such as cola soft drink was a social differentiator as a symbol of prestige. Similarly, in the present study, women with HBMI indicated being proud of overeating, or eating foods other than beans or edible cactus, which are mostly consumed by underprivileged people, which shows that they enjoy a good life. The social theory of food as a distinction in each SES group was documented many years ago by Bourdieu [33]. His research showed that in lower SESs, people are more eager to take advantage of good moments such as enjoying food, and since their future may seem unfortunate for them, they seek to experience a good life in the immediate present. In this study, women with HBMI also seemed to focus on enjoying life in the short term instead of avoiding health consequences to ensure a happy life in the long term.

### Body size meanings

An important finding in the study was that overweight was socially accepted in children and married women. It has been documented that, in Mexico, as well as in other countries, those who decide to live with their partner increase their BMI more than those remaining single [34–36]. The theories presented in this study may help to explain the rationale for overweight or obesity acceptance among the studied medium-low SES women living with their partners. Among the studied women, being fat was a symbol of enjoying a good life. This was elucidated by analyzing negative comments about normal-weight children or women related to undernourishment, mistreatment, or disease. Moreover, several comments revealed that an overweight or obese woman who pursues a normal weight may seem like she is trying to look appealing for men, which was not socially accepted. In addition, women may feel more relaxed about their weight, since they are no longer in the ‘marriage market;’ this theory was already proposed by Averett [37] regarding a U.S. population. The belief of good health associated with overweight is especially notable when women are lactating, since women avoid weight loss after pregnancy by overeating and even stopping breastfeeding.

### Motivation to improve eating behavior

The present study revealed that women with HBMI, although they may disapprove of their weight, did not worry enough to make a definite change unless they perceived a physical indication of poor health or had a fear of dying. Thus, overweight may be socially accepted in middle-low socioeconomic stratum groups, especially in the rural area. Findings similar to this result were described by Christakis and Fowler [38], who named it “contagious obesity.” Their findings suggested that social norms and behaviors that are associated with obesity spread through social networks. For instance, the motivation to lose weight or improve eating habits might be suppressed by the presence of other antagonist social norms such as the social prestige of overeating or being overweight. Therefore, future strategies to motivate healthy eating in order to decrease obesity and its co-morbidities, should utilize messages that support rather than oppose social norms and beliefs. Examples of possible public health messages targeting the studied population are: encouraging the pursuit of a strong body through healthy eating rather than the pursuit of a slim body, recommending the consumption of healthy food for enjoyment rather than in order to attain good health, or promoting healthy behavior so that one may be living when one’s children grow up, rather than in order to be healthy.

## Conclusion

The present study proposes possible sociocultural causes of inadequate eating habits that may lead to obesity. Women with HBMI from medium-low SESs in rural as well as urban areas share strong beliefs and social norms about eating and body size, in opposition to behaviors related to healthy eating and achieving a normal BMI. Due to the fact that the study was qualitative, the conclusions cannot be generalized to a certain population; thus, studies are needed in order to confirm the social factors elucidated in this study, in the same population, and also to compare them with other populations such as different SES groups, men, and children.

Our findings provide evidence that certain core beliefs and social norms are important determinants of eating behavior. The fact that sociocultural factors have not been taken into account could be the reason why anti-obesity strategies have not been as effective as was expected. The belief system of particular social groups within different SESs should be considered in order to understand the etiology of obesity and to develop effective strategies.

## Abbreviations

HBMI: High body mass index
RTEC: Ready to eat cereal
SES: Socioeconomic status

## Footnotes

1 *Tortilla* (a staple food in Mexico), is a thin, round, unleavened bread prepared from cornmeal, baked on a flat plate of iron.

2 *Nopales* are a type of edible cactus (*Opunita sp*).

3 *Tacos* are tortillas filled with food: beans, meat, or vegetables.
